# HER2 Oncogene Amplification and Immunohistochemical Profiling in Gastric Adenocarcinoma

**DOI:** 10.15190/d.2018.6

**Published:** 2018-12-31

**Authors:** Nisha Raj, Divya Verma, Ashok Kumar, Praveer Rai, Ram Nawal Rao

**Affiliations:** Department of Pathology, Sanjay Gandhi Post Graduate Institute of Medical Sciences, Lucknow, India; Department of Surgical Gastroenterology, Sanjay Gandhi Post Graduate Institute of Medical Sciences, Lucknow, India; Department of Gastroenterology, Sanjay Gandhi Post Graduate Institute of Medical Sciences, Lucknow, India

**Keywords:** Gastric adenocarcinoma, Immunohistochemistry, Fluorescence in situ hybridization, Human Epidermal Growth Factor Receptor 2, HER-2, Haematoxylin and Eosin.

## Abstract

* Background and Objectives: * Gastric adenocarcinoma is one of the most common malignant tumors and a major cause of cancer death worldwide, especially in developing countries. Her2/neu gene amplification and protein overexpression in breast cancer is a golden criterion for the targeted therapy with trastuzumab. However, the role of Her2 as a prognostic factor in gastric cancer is still controversial. The purpose of this study was to evaluate the frequency of Her2 oncogene overexpression and concordance between the results for Her2 protein expression and gene amplification. *Materials and Methods: * A total of 65 retroprospective cases with gastric adenocarcinoma, including biopsy and resected specimens obtained between July 2015 to December 2017, were analyzed. Her2/neu expression was determined by Immuno-histochemistry (IHC). Equivocal and some selected cases were submitted for FISH to detect Her2/neu gene amplification. *Results:* In the present study, out of 65 patients of gastric adenocarcinoma, there were 50 males and 15 females, with mean age of 54.52 years. The majority of tumors were located within the antropyloric region. We found 27 (41.4%) positivity, scored as IHC 3+ and IHC 2+, and 38 (58.3%) negativity, scored as IHC 1+ and IHC 0. We also evidentiated a significant difference between Her2/neu expression with age (p=0.010) and depth of invasion (p=0.020).Her2/neu gene was amplified only in 13 cases, 4 cases were of Her2/neu (3+) positive, 11 cases (39.3%) Her2/neu (2+) with IHC staining. The concordance rate between the results of IHC and FISH in all 18 cases was 83.3%. *Conclusion:* IHC detection can be carried out to guide the treatment when FISH detection cannot be performed. Overexpression of Her 2/neu in gastric adenocarcinoma could potentially be used in selecting the patients who can get benefit from the anti-Her2/neu targeted therapy.

## **INTRODUCTION**

Gastric cancer remains one of the most prevalent malignant tumors^1,2^ and it represents one of the major causes of cancer related mortality worldwide. Adenocarcinoma is one of the most common histological types of gastric cancer. Incidence of gastric cancer varies widely among the various regions worldwide and within India, due to the diverse ethnic groups and the related food habits. Reports from the National Cancer Registry Programme (NCRP) 2010 suggested that the mean age-adjusted rate (AAR) of gastric cancer among urban registries in India varied from 3.0 to 13.2, with the highest rate being recorded in Chennai registry^3-5^. However, the prevalence was found to be much higher in the Northeastern region of India. In India, the age range for stomach cancer is 35-55 years in the South and 45-55 years in the North. The disease shows a male preponderance in almost all countries, with rates two to four times higher among males than in females^6-8^.

Carcinoma of the stomach is assumed to originate from a sequential accumulation of molecular and genetic alterations to stomach epithelial cells, but the mechanism of carcinogenesis remains complex and poorly understood^9-11^. The search for cancer biomarkers is carried out in order to identify tumor cells at early stages and predict treatment response, ultimately leading to a favorable therapeutic outcome.

Human epidermal growth factor receptor (Her2/neu) has tyrosine kinase activity and is a member of the epidermal growth factor receptor family. Her2/neu gene is a proto-oncogene which is located on the chromosome 17q21. It acts as an oncogene due to its amplification, which leads to the overexpression of Her2/neu protein^12,13^.

Her2/neu protein exists as a monomer. After ligand binding, these monomers form receptor dimers, which can either be homodimers with the same receptor type (e.g. Her1-Her1) or heterodimers with different receptor types (e.g. Her1-Her2). Dimerization of the receptor results in the autophosphorylation of the tyrosine residues within the cytoplasmic domain of the receptors, and initiates a variety of signaling pathways, leading to cell proliferation and tumorigenesis.

Herceptin’s (trastuzumab) use in breast cancer highlights the importance of an appropriate Her2/neu evaluation to ensure accurate identification of patients eligible for the anti-Her2 targeted therapies in gastric cancer.

Her2/neu expression rate in gastric and gastroesophageal junction carcinomas has been detected to vary from 4.4% to 53.4%^14-24^, while for the amplification of gene copy number by FISH it varies from 1% to 42.6%^25-28^. In India, data regarding Her2/neu status in gastric cancer is available in only a limited number of studies^13,29,30^. Determination of Her2/neu’s prognostic value is still controversial, with some studies identifying it as a negative prognostic factor for survival, some as positive prognostic factor for survival, and others failing to find a relationship^26-28,31,32^. The relationship between the level of Her2/neu amplification and the outcome of Her2/neu-positive gastric cancer treated with first-line chemotherapy with trastuzumab remains unclear.

An accurate assessment of Her2/neu status is essential in selecting the patients who would most benefit from the targeted therapy with trastuzumab, among the patients with gastric cancer. The purpose of this study was to evaluate the frequency of Her2/neu protein overexpression and the concordance between Her2/neu protein expression and the gene amplification in gastric adenocarcinomas. 

## **M**ATERIALS AND METHODS

### *Patient Specimens*

We reviewed the tissue obtained from both biopsy and gastrectomy samples in 65 patients of gastric and gastroesophageal junction adenocarcinomas, at the Sanjay Gandhi Postgraduate Institute of Medical Sciences, in Lucknow, India, from 2015 to 2017, in accordance with internal protocols and standards.

The clinical and demographic features were retrieved from the medical records file of each patient and from hospital information system. The patient’s age was divided into two groups (under and above 55 years old). The diagnosis was established with the histopathological evaluation of endoscopic biopsy or surgical resection material. The tumors were evaluated histologically as follows: well-differentiated tumors included grade I and moderately differentiated tumors grade II adenocarcinomas; poorly differentiated on the other hand, consisted of grade III adenocarcinomas.

### *Immunohistochemistry*

All samples were fixed in 10% neutral buffered formalin, embedded in paraffin, cut in 3-5 μm sections and stained with hematoxylin & eosin for histological typing and grading. Blocks of biopsy specimens were also evaluated for adequacy of tumor tissue for IHC. Immunohistochemistry (IHC) was performed on freshly cut deparaffinized sections using the peroxidase-labelled streptavidin-biotin technique. Her2/neu detection was performed with heat induced epitope retrieval (HIER) citrate buffer (Target Retrieval Solution, pH 6.1, Dako) in a water bath at 98℃. Following incubation with the anti-Her2neu primary antibody (Polyclonal Rabbit), the visualization is based on the sequential application of streptavidin-horseradish peroxidase conjugate secondary antibody (Dako, Los Angeles, CA, USA). Subsequently added chromogen results in a visible reaction product at the antigen site. As a positive control, breast cancer tissue having strong membranous immunostaining (score 3+) was concomitantly stained. Slides were counterstained with hematoxylin for 3-5 min. Her2/neu immunohistochemistry slides were scored by two pathologists individually, using scoring guidelines proposed by Hofmann et al. and Ruschoff et al.^33,34^**(Table 1)**.

**Table 1:** Immunohistochemistry scoring criteria for Her2/neu in gastric adenocarcinoma

IHC Score	Her2 protein overexpression	Staining pattern (surgical specimen)
0	Negative	No membranous reactivity in <10% any tumor cells.
1+	Negative	Faint or barely perceptible membranous reactivity in ≥10% of tumor cells.
2+	Equivocal	Weak to moderate complete, basolateral or lateral membranous reactivity in ≥10% of tumor cells.
3+	Positive	Strong complete, basolateral or lateral membranous reactivity in ≥10% of tumor cells.

### *Fluorescence in situ Hybridization*

Specimens with IHC 2+ scores/equivocal cases were tested using fluorescent in situ hybridization (FISH). For each case, successive sections from the same block were used to compare IHC with FISH results. FISH analysis was carried out according to the manufacturer’s protocol, by using the PathVysion^TM^ Her2 DNA Probe detection kit (Abbott, Illinois, USA, LSI-Her2/neu Spectrum Orange^TM^/ chromosome 7 centromere probe (CEP) VR 17 Spectrum Green^TM^). FISH positive was defined as Her2:CEP17 ratio ≥2. Her2 scoring was done in accordance with CAP/ASCO guidelines^35,36^. The presence of gene amplification was determined using the fluorescence-labelled DNA probe for chromosomal locus 17q11.2-q12, for the detection of Her2/neu. 

### *Interpretation of FISH*

FISH-stained sections were scanned at × 1000 magnification and in each of three separate carcinoma areas. For the FISH analyses a minimum of 20-40 cells were counted and a ratio of Her2/neu to CEP17 ratio greater than 2 was interpreted as positive for gene amplification, a ratio less than 1.8 as negative, and ratio between 1.8 to 2 as equivocal.

### *Statistical Analysis*

The obtained results were analyzed by statistical software SPSS (IBM SPSS Statistics 20). Chi-square and Fisher's exact tests were used to analyze the correlation between the expression of Her2/neu in gastric adenocarcinomas and clinicopathological features. A p-value less than 0.05 was considered statistically significant. Different diagnostic parameters (sensitivity, specificity, positive predictive value, negative predictive value) for predicting FISH-confirmed Her2/neu status were calculated, and the two methods were compared using 95% confidence interval. In addition, kappa statistic was calculated for assessing the concordance between the methods.

## RESULTS

A total of 65 patients (50 men and 15 women) were enrolled and evaluated in this study. The mean age of the patients was 54.52 years. By anatomic site, most of the tumors were located in antropyloric region 38 (58.4%). Moderately differentiated adenocarcinoma was predominant, being found in 30 patients (46.1%), followed by the poorly differentiated adenocarcinoma in 23 patients (35.3%) and well differentiated adenocarcinoma type in 12 patients (19%).

According to the Lauren’s classification, maximum number of cases were of intestinal type: 38 (58.4%). The clinicopathological findings in patients are summarized in **Table 2**.

**Table 2:** Correlation of Her2/neu status with patient information and characteristics by immunohistochemistry

Clinical Variables	N=65	Her2 IHC Score Positive	Her2 IHC Score Negative	p-value
Gender				0.544
Male	50	22	28	
Female	15	5	10	
Age				0.010
≤55	36	10	26	
>55	29	17	12	
Tumor site				0.893
Gastric	54	23	31	
GE-Junction	11	4	7	
Lauren Classification				0.516
Intestinal	36	13	23	
Diffuse	24	11	13	
Mixed type	5	3	2	
Tumor Differentiation				0.918
Grade I	13	6	7	
Grade II	29	12	17	
Grade III	23	9	14	
Depth of Invasion				0.020
Yes	36	20	16	
No	29	7	22	
Lymph node metastasis				0.134
Yes	42	19	23	
No	23	8	15	

### *Her2/neu Protein Overexpression by IHC*

**Figure 1:**
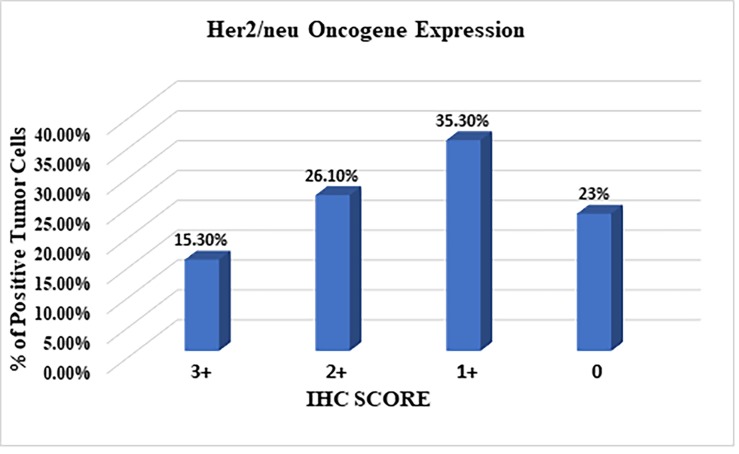
Frequency of Her2/neu expression in gastric adenocarcinomas

**Figure 2:**
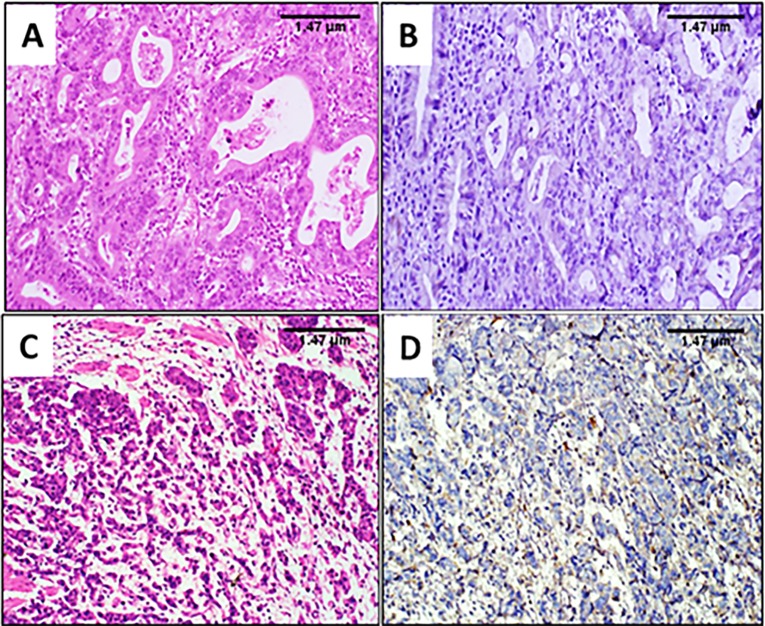
A-B. H&E of moderately differentiated adenocarcinoma and corresponding IHC showing no Her2 staining on tumor cell (score 0, negative). C-D. H&E of signet ring cell and corresponding IHC showing faint, barely perceptible Her2 staining (score 1+, negative).

**Figure 3:**
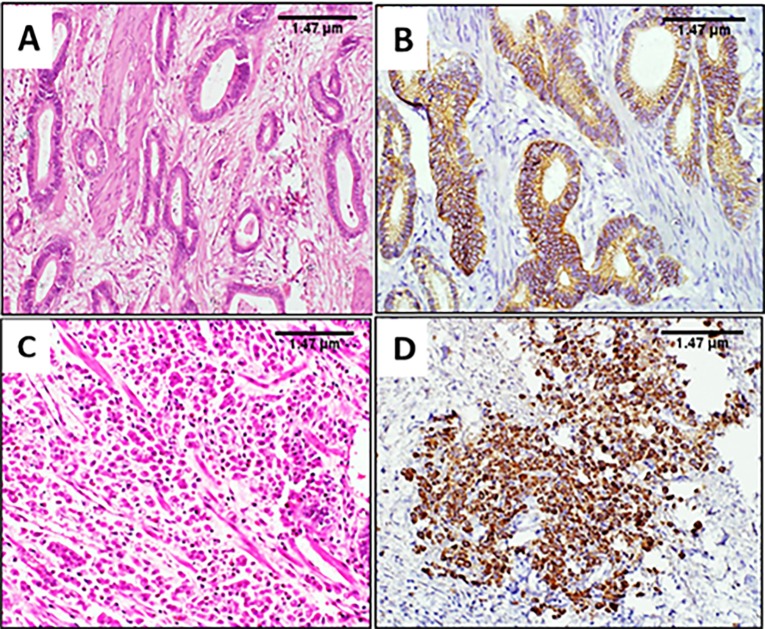
A-B. H&E of well differentiated adenocarcinoma and corresponding IHC showing moderate complete membranous Her2 staining in glands (score 2+, positive). B-C. H&E of signet ring cell adenocarcinoma and corresponding IHC showing strong Her2 immunostaining (score 3+, positive).

Her2/neu immunostaining was performed in 65 of the cases of gastric adenocarcinomas. 10 out of 65 (15.3%) cases were scored as 3+ (positive), 17 (26.1%) cases were scored as 2+ (equivocal) and 38 (58.3%) cases as 1+ and 0 (negative) **(Figure 1)**. Higher Her2/neu positivity was reported in the cases with intestinal type of adenocarcinoma, 13 (48.1%), followed by the diffuse type, 11 (40.7%), and the mixed type, 3 (11.1%) **(Table 3, Figure 2, Figure 3)**. 

**Table 3:** Correlation of Her2/neu expression with Lauren classification

Laurens Classification	Total number of patients	Her2 IHC Score 0	Her2 IHC Score 1+	Her2 IHC Score 2+	Her2 IHC Score 3+
Intestinal type	36	7	16	8	5
Diffuse type	24	6	7	7	4
Mixed type	5	2	0	2	1
Total	65	15	23	17	10

Likewise, frequency of Her2/neu overexpression was higher in moderately differentiated adenocarcinoma, 12 cases (44.4%) (**Table 4**)**.**

**Table 4:** Correlation of Her2/neu oncogene with histological grade differentiation

Laurens Classification	Total number of patients	Her2 IHC Score 0	Her2 IHC Score 1+	Her2 IHC Score 2+	Her2 IHC Score 3+
Well differentiated	13	4	3	4	2
Moderately differentiated	29	7	10	7	5
Poorly differentiated	23	6	8	5	4
Total	65	17	21	16	11

By comparing Her2/neu expression with clinicopathological features in patients with gastric adenocarcinoma and statistical analysis of data, a significant correlation was not found in between the expression of tumor marker, gender, tumor location, Lauren classification, histologic grade and the number of involved lymph nodes (p>0.05). However, our results showed significant difference in Her2/neu expression with age (p=0.010) and depth of invasion (p=0.020) **(Table 2). **

### *Her2/neu gene amplification by* *FISH*

In this study, out of the 65 cases of gastric adenocarcinoma, FISH was performed only in 18 cases. Four of 18 cases showed gene amplification having Her2/CEP ratio >2. Nine cases showed equivocal amplification, having Her2/CEP ratio between 1.8-2 and five cases had no gene amplification, with a ratio <1.8.

### *Concordance between IHC and FISH*

**Figure 4:**
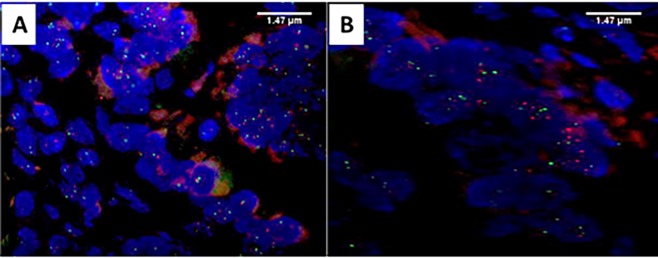
Fluorescence in situ hybridization using probes directed against Her2/neu in gastric adenocarcinoma (FISH, Her2 gene x 1000). A. Case of moderately differentiated adenocarcinoma negative for Her2 gene amplification, IHC Score 2+, Red signals (Her2/neu gene), green signals (chromosome enumeration probe 17 (CEP17)), blue signals (nuclei stained with DAPI) having Her2/CEP ratio between 1.8-2. B. Case of well differentiated adenocarcinoma, positive for Her2/neu gene amplification, showing red clusters (orange arrow) and was, IHC score 3+, having Her2/CEP ratio >2.

When the results of FISH and IHC were compared, among the 11 tumors with 2+ (equivocal) immunostaining only 9 showed amplification, while within the 5 tumors with 3+ strong membranous immunostaining, 4 showed Her2/neu amplification (**Figure 4**). Among the 2 cases with score 0 or 1+, evidencing negative immunostaining, none exhibited amplification. For two cases, precise quantitation was proven to be impossible, showing one or two large clusters of tightly coalesced signals. However, the image analysis of each cluster showed more than 10-fold the fluorescence of the single-copy signals. Five tumors exhibited no amplification and 13 tumors exhibited amplification. In our study, we found a strong correlation between protein expression and gene amplification by FISH (p=0.001), as shown in **Table 5**. Her2/neu protein expression and gene amplification levels were found to be linearly associated and highly concordant.

**Table 5:** Concordance between Her/neu oncogene amplification and its protein overexpression Cross tables; Pearson Chi- Square test; p value = 0.001 (highly significant); IHC: Her2/neu protein expression;

Her2/neu Gene amplification	IHC Negative	IHC Equivocal	IHC Positive	Concordance
FISH(+)	0	9	4	13 (100%)
FISH(-)	2	2	1	5 (40%)
Total	2	11	5	18 (83.3%)

### *Sensitivity, Specificity and overall Accuracy between IHC and FISH*

The sensitivity and specificity of Her2/neu IHC were evaluated, using FISH as the standard test. We found 100% sensitivity, 40% specificity, with a positive predictive value of 81.25% and negative predictive value of 100%, as shown in the **Table 6**. This study revealed a good agreement (k=0.692, p=0.001) between IHC and FISH, with overall accuracy of 83.3%.

**Table 6:** Diagnostic performances of Her2/neu Gene Amplification in relation toHer2/neu protein expression

Parameters	Between FISH and IHC
Sensitivity	100%
Specificity	40%
Positive predictive value (PPV)	81.25%
Negative predictive value (NPV)	100%
Overall accuracy	83.3%

## DISCUSSION

Stomach carcinogenesis is a multistep process involving multiple genetic and epigenetic alterations in oncogenes, tumor suppressor genes, DNA repair genes, cell cycle regulators and signaling molecules^37,38^.

In gastric adenocarcinomas, the prevalence rates of Her2/neu expression range from 10% to 22.8%^14^. Her2/neu protein overexpression and gene amplification has been detected in 15% to 20% of patients with gastric and gastroesophageal junction cancer^39^. Trastuzumab, a monoclonal antibody anti-Her2/neu protein, is a promising agent for the treatment of patients with Her2/neu overexpressing breast cancer, and has also been found to exhibit antitumor activity in human gastric cancer cells that overexpress Her2/neu. These targeted drugs selectively act on cancer cells at the molecular level, specifically targeting abnormal cells, with minimal effects on the function of normal cells. Therefore, an accurate evaluation of Her2/neu status in gastric and gastroesophageal junction adenocarcinoma has become increasingly important^40^. 

In this study, intestinal-type adenocarcinoma accounts for 58.4 % of our cases, which is similar to the previous reported data^41^. Frequency of gastric cancer in intestinal type is varies from 6.1% to 28.57% and in the diffuse type from 0.7% to 13.43%. Ahmed Abdel Hadi et.al.^42^ reported that, the majority of cases belong to moderately differentiated tumors, which is similar to our study (46.1%). However, our results contrast to another study, which found 58.1% of cases belonging to tumors with poorly differentiated adenocarcinoma^41^.

The most widely used methods to evaluate Her2/neu status are IHC and FISH. Compared to IHC, the FISH method is more standardized and less variable. Therefore, FISH emerged as a “gold standard” method for the assessment of Her2/neu status^43,44^. Immunohistochemistry is a semi-quantitative method, easier to perform, relative inexpensive and is used more often. However, the sensitivity and specificity of the assay can vary significantly, depending on the commercial antibody used. 

Various literature reported that in gastric cancer patients the overexpression rate of Her2/neu gene was 5.2% to 22.6%, and the amplification rate of Her2/neu gene was 3.8% to 12.2% by FISH detection^21,45,46^. In agreement with the Indian study by Shekharan A et al.^29^, the frequency of Her2/neu protein expression was 41.1%. Another study in India performed by Lakshmi V et al.^47^ reported Her2/neu positivity of 35.9%. Our previous study^48^ reported that the percentage of Her2/neu expression was higher in moderately differentiated adenocarcinoma as compared to the well and poorly differentiated adenocarcinoma. However, this correlation was not statistically significant (p=0.945), which is consistent with this and other studies^16,49-52^.

Several studies pointed out the significant association between Her2/neu protein overexpression and tumor differentiation^39,53^, while others do not find this association^46,54^. These conflicting data may be due to different sample sizes and low prevalence of Her2/neu in GAC and gastroesophageal junction adenocarcinoma.

Similar to the studies by Sutapa Halder et.al.^49^ and De Carli DM et.al.^55^, intestinal type showed the most Her2/neu protein expression (48.1%), followed by diffuse and mixed type adenocarcinoma. We didn’t find any significant association between Her2/neu expression with gender, location of tumor, Lauren’s classification, degree of differentiation and Lymph node metastasis^14,52,54,56,57^ (p>0.05). However, our results showed significant difference in Her2/neu expression with age (p=0.010) and depth of invasion (p=0.020).

Rate of Her2/neu gene amplification was 72.2%, which is in accordance to Yan Song et.al^58^ and Yano et.al.’s^59^ findings. These results were higher than those stated in different studies^21,45,46^. These high rates of Her2/neu gene amplification may be due to the malignant progression of gastric adenocarcinoma to some extent.

The status of protein expression is not absolutely consistent with that of gene amplification, especially in IHC 3+ and IHC 2+ cases. The cases without gene amplification may have protein overexpression, while the cases with high gene amplification may have low or no protein expression. We found that 3 of 16 cases with protein expression exhibited no gene amplification. This discrepancy may be associated with polyploidy of chromosome 17, fragment length of Her2/neu mRNA untranslated region (UTR), post-transcriptional regulation and IHC false positive results. It can be speculated that post-transcriptional or post-translational regulation of Her2/neu gene probably plays a certain role in gastric adenocarcinoma. In our research, there was a high concordance between IHC and FISH (83.3%) and a significant association between Her2/neu IHC and FISH (p=0.001)^40,42,54,60^.

Published studies investigating Her2/neu expression in GAC have been very small in size, never been validated in a second independent set and therefore, results regarding the relationship of Her2/neu expression and clinicopathological data including patient survival are still controversial.

### *Limitations*

Intratumoral heterogeneity and small number of patients are factors that make it difficult to evaluate the prognostic significance of Her2/neu protein. Interpretation of both IHC 1+ and 2+ cases by FISH is challenging in small biopsies due to crush and inadequacy of sample, and also because of standardized scoring criteria.

## **CONCLUSION**

Due to high concordance between Her2/neu gene amplification and protein overexpression, especially in IHC 3+ patients, IHC detection can also be carried out to guide the treatment when FISH detection cannot be performed. Further research is also required to explore Her2/neu protein targeted therapies, such as trastuzumab (Herceptin), for gastric adenocarcinoma. However, there is still a need for a unique and more precise scoring system in FISH and IHC interpretations, potentially by using advanced machine learning-based image analysis techniques.
